# Nitration of the Egg-Allergen Ovalbumin Enhances Protein Allergenicity but Reduces the Risk for Oral Sensitization in a Murine Model of Food Allergy

**DOI:** 10.1371/journal.pone.0014210

**Published:** 2010-12-02

**Authors:** Eva Untersmayr, Susanne C. Diesner, Gertie Janneke Oostingh, Kathrin Selzle, Tobias Pfaller, Cornelia Schultz, Yingyi Zhang, Durga Krishnamurthy, Philipp Starkl, Regina Knittelfelder, Elisabeth Förster-Waldl, Arnold Pollak, Otto Scheiner, Ulrich Pöschl, Erika Jensen-Jarolim, Albert Duschl

**Affiliations:** 1 Department of Pathophysiology and Allergy Research, Center of Pathophysiology, Infectiology and Immunology, Medical University of Vienna, Vienna, Austria; 2 Department of Pediatrics and Adolescent Medicine, Medical University of Vienna, Vienna, Austria; 3 Department of Molecular Biology, University of Salzburg, Salzburg, Austria; 4 Biogeochemistry Department, Max Planck Institute for Chemistry, Mainz, Germany; Dana-Farber Cancer Institute, United States of America

## Abstract

**Background:**

Nitration of proteins on tyrosine residues, which can occur due to polluted air under “summer smog” conditions, has been shown to increase the allergic potential of allergens. Since nitration of tyrosine residues is also observed during inflammatory responses, this modification could directly influence protein immunogenicity and might therefore contribute to food allergy induction. In the current study we have analyzed the impact of protein nitration on sensitization via the oral route.

**Methodology/Principal Findings:**

BALB/c mice were immunized intragastrically by feeding untreated ovalbumin (OVA), sham-nitrated ovalbumin (snOVA) or nitrated ovalbumin (nOVA) with or without concomitant acid-suppression. To analyze the impact of the sensitization route, the allergens were also injected intraperitoneally. Animals being fed OVA or snOVA under acid-suppressive medication developed significantly elevated levels of IgE, and increased titers of specific IgG1 and IgG2a antibodies. Interestingly, oral immunizations of nOVA under anti-acid treatment did not result in IgG and IgE formation. In contrast, intraperitoneal immunization induced high levels of OVA specific IgE, which were significantly increased in the group that received nOVA by injection. Furthermore, nOVA triggered significantly enhanced mediator release from RBL cells passively sensitized with sera from allergic mice. Gastric digestion experiments demonstrated protein nitration to interfere with protein stability as nOVA was easily degraded, whereas OVA and snOVA remained stable up to 120 min. Additionally, HPLC-chip-MS/MS analysis showed that one tyrosine residue (Y_107_) being very efficiently nitrated is part of an ovalbumin epitope recognized exclusively after oral sensitization.

**Conclusions/Significance:**

These data indicated that despite the enhanced triggering capacity in existing allergy, nitration of OVA may be associated with a reduced *de novo* sensitizing capability via the oral route due to enhanced protein digestibility and/or changes in antibody epitopes.

## Introduction

The prevalence of type I allergies is steadily increasing in Western societies [1]. A reduced exposure to microbial pathogens in childhood has been discussed to represent one of the major causes, summarized in the so-called hygiene hypothesis [2]. However, additional risk factors including individual (e.g. nutrition and medication) as well as environmental factors play an essential role in allergy induction.

In the last decades, ambient pollutants, combustion generated particles and polycyclic aromatic hydrocarbons associated with them have been identified as health problems with substantial impact on our immune responses [3]–[5]. The components of smog, NO_2_ and O_3_, have been discussed to chemically alter airborne proteins, resulting in e.g. protein nitration. This effect has been demonstrated for Bet v1, the major birch pollen allergen, with was detected in nitrated form in dust samples from various urban environments [6]. Nitration of protein tyrosine residues, resulting in 3-nitrotyrosine formation, changes the conformation of proteins, being of special importance for B-cell epitopes and may, thus, alter not only protein function but also IgE recognition substantially [7]. Slight modifications of amino acids within proteins by nitration can increase or diminish the potential of epitope binding to immunoglobulins. Even though the dramatic effect of some point mutations on IgE binding has been elucidated using Bet v1 as a model allergen [8], [9], nitration has been discussed to especially alter IgG epitopes [10]. However, a recent study has revealed that the allergenic potential of Bet v1 for inducing IgE-dependent type 1 allergy is also increased by protein nitration [11]. Additionally, nitration of Bet v1 was demonstrated to be associated with an enhanced antigen processing and presentation by dendritic cells leading to a stronger stimulation of Bet v1 specific T cells (Karle AC et al. Unpublished data).

While tyrosine nitration due to environmental air pollutants was noted only recently, protein nitration has previously been described to occur within inflammatory conditions and as a result of the ageing process in the human body [12]. Nitration of tyrosine residues from self-antigens may promote the development of autoimmune reactions by evasion of immunological self-tolerance [13]. Additionally, protein nitration can occur in situations like inflammatory bowel diseases or Helicobacter (H.) pylori infections [14], [15]. Under these circumstances, common proteins of the daily diet might undergo modification by nitration with major impact on immunogenicity and consequently also on food allergy induction.

Already in the 1980ies, the association between gastrointestinal inflammations and a higher incidence of food allergy has been reported, with food allergic reactions being attributed to hereditary and alimentary factors [16]. Later, an association between H. pylori infection and food allergy was reported [17]–[19] and high levels of total and food-specific IgE were measured in the gastrointestinal mucosa of peptic ulcer patients [20], [21]. On the one hand the correlation of gastric ulcers and food allergy was discussed as a result of enhanced mucosal permeability in H. pylori infection [22]; on the other hand the current treatment of dyspeptic disorders with acid-suppression medication was revealed as a causative factor for IgE formation [23]–[25].

It is conceivable that protein nitration induced during inflammatory responses in the stomach could directly influence protein immunogenicity and thus contribute to food allergy induction. In the present study we aimed to investigate the influence of protein nitration on allergy induction via the oral route using a previously established murine food allergy model [23], [26].

## Results

### Characteristics of nOVA proteins

#### Spectrophotometric analysis of nOVA samples revealed a 21% nitration grade of the 10 tyrosine residues

Additionally, we investigated the location of nitrated tyrosine residues within the nOVA protein and evaluated the nitration degree of the tyrosine residues by HPLC-chip-MS/MS analysis. Native OVA consists of 386 amino acid residues including 10 tyrosine residues. In the HPLC-chip-MS/MS analysis of nOVA we could identify 11 tryptic peptides and achieved an amino acid coverage of 33%. Three tyrosine residues could be located in native (Y_112_, Y_118_ and Y_282_) and nitrated (Y_107_, Y_112_ and Y_282_) form (Table 1).

**Table 1:** HPLC-chip-MS/MS-analysis.

Position	Sequence	RT(min)	z	m/z(Da)	m_t_(Da)	nativetyrosine	nitrated tyrosine	NDY^a^
**105–112**	(R)LYAEER(Y)	1.13	2	413.19	780.39		Y_107_	1
**111–124**	(R)YPILPEYLQCVK(E)	14.10	2	761.90	1522.80	Y_112_; Y_118_		
**111–124**	(R)YPILPEYLQCVK(E)	15.19	2	784.80	1522.80	Y_118_	Y_112_	0.11±0.08
**127–144**	(R)GGLEPINFQTAADQAR(E)	11.96	3	844.42	1687.84			
**143–160**	(R)ELINSWVESQTNGIIR(N)	14.48	2	929.99	1858.97			
**187–201**	(K)AFKDEDTQAMPFR(V)	1.45	3	519.24	1555.72			
**190–201**	(K)DEDTQAMPFR(V)	1.53	2	605.26	1209.52			
**264–278**	(K)LTEWTSSNVMEER(K)	10.02	2	791.36	1581.72			
**280–286**	(K)VYLPR(M)	1.13	2	324.20	647.39	Y_282_		
**280–286**	(K)VYLPR(M)	1.46	2	346.69	647.39		Y_282_	0.14±0.19
**323–341**	(K)ISQAVHAAHAEINEAGR(E)	1.09	2	887.45	1773.90			
**340–361**	(R)EVVGSAEAGVDAASVSEEFR(A)	11.84	2	1004.98	2008.95			
**370–383**	(K)HIATNAVLFFGR(C)	13.39	3	449.25	1345.74			
**370–383**	(K)HIATNAVLFFGR(C)	13.39	2	673.37	1345.74			

a NDY =  I*_nitrated_*/(I*_nitrated_*+I*_native_*); I (Intensity).

Identified tryptic peptides of nOVA: amino acid sequence, retention time (RT), measured mass-to-charge ratio (m/z), charge stage (z), theoretical molecular mass (m_t_) tyrosine residue and calculated nitration degrees of individual tyrosine residues (NDY).

#### Oral immunizations result in elevated titers of IgG1 and IgG2a when OVA or snOVA are fed under acid-suppression

**Figure 1:**
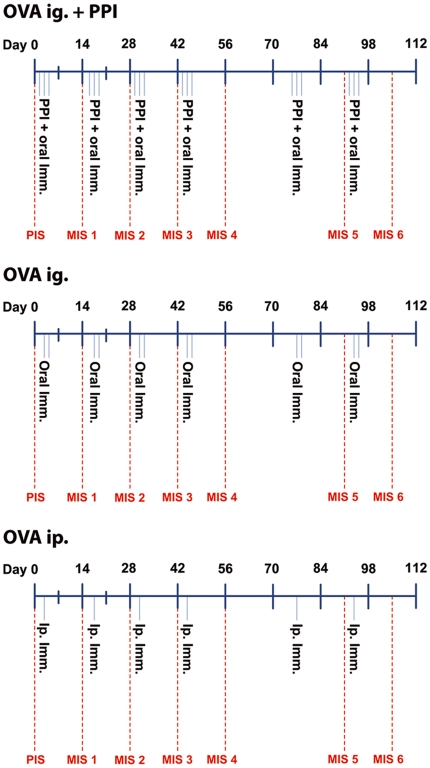
Flow diagram of the applied immunization protocol. Animals divided in 10 groups (n = 6) were repeatedly immunized with OVA, snOVA or nOVA either with or without concomitant gastric acid-suppression. Furthermore, 3 groups were injected the respective allergens ip. A control group remained naïve. Blood for measurement of antibody titers was collected on days 0, 14, 28, 42, 56, 91 and 105.

**Figure 2:**
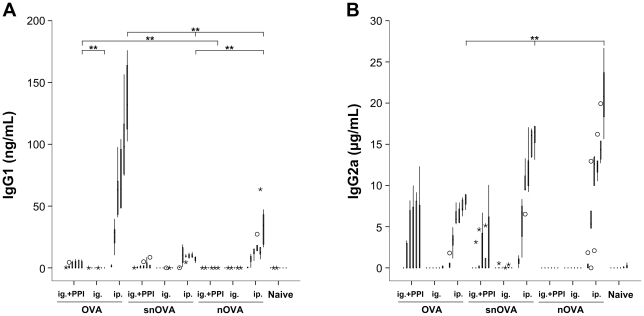
Elevated titers of OVA-specific IgG1 and IgG2a antibodies are observed after oral immunizations with OVA and snOVA under gastric acid suppression. (A) Increased levels of OVA-specific IgG1 antibodies are detected in the gastric acid suppressed animals receiving OVA or snOVA ig. as well as in the groups immunized ip. with OVA, snOVA and nOVA. Animals of groups fed with OVA, snOVA or nOVA alone or nOVA under gastric acid suppression and the naïve animals revealed only marginal or undetectable IgG1 titers. (B) Specific IgG2a antibodies are also detected only in animals immunized with OVA or snOVA ig. under concomitant gastric acid suppression as well as in the ip. injected groups. In the other groups the levels remained at the baseline. The boxes represent the inner quartiles value range of the six immune sera with the median indicated. Sera with signals more than 1.5-fold interquartile range deviation from the end of the box were defined as outliers and marked as circles. Sera with titers lying more than 3-fold interquartile range away were defined as extremes and marked with asterisks. Brackets indicate the groups with statistical significant differences in antibody titers (**P<0.01).

After 6 immunization cycles with OVA, snOVA or nOVA (Figure 1, Table 2) enzyme-linked immuno sorbent assay (ELISA) screening of OVA-specific antibodies in murine serum samples demonstrated an increase of OVA-specific IgG1 only in the animals being fed OVA and snOVA under concomitant gastric acid suppression. All other animals immunized ig., including those being fed nOVA with or without anti-acid medication, did not develop detectable levels of specific IgG1 antibodies during the time course of repeated immunization (Figure 2A). Accordingly, IgG2a was only elevated in the groups being orally immunized either with OVA or snOVA after six immunization cycles (Figure 2B). Analyzing the impact of the route of sensitization, the 3 groups immunized intraperitoneally (ip.) with OVA, snOVA or nOVA revealed elevated levels of OVA-specific IgG1 and IgG2a (Figure 2A, B).

**Table 2:** Immuization protocol.

Group label	Immunized with
**OVA ig. + PPI**	116 µg PPI, 200 µg OVA +2 mg sucralfate ig.
**OVA ig.**	200((g OVA ig.
**OVA ip.**	2((g OVA +2% Al(OH)3 ip.
**snOVA ig. + PPI**	116 µg PPI, 200 µg snOVA +2 mg sucralfate ig.
**snOVA ig.**	200 µg snOVA ig.
**snOVA ip.**	2 µg snOVA +2% Al(OH)_3_ ip.
**nOVA ig. + PPI**	116 µg PPI, 200 µg nOVA +2 mg sucralfate ig.
**nOVA ig.**	200((g nOVA ig.
**nOVA ip.**	2((g nOVA +2% Al(OH)3 ip.
**Naive**	—

#### Functional OVA-specific IgE antibodies are detected in animals immunized orally with OVA or snOVA under acid-suppression

**Figure 3:**
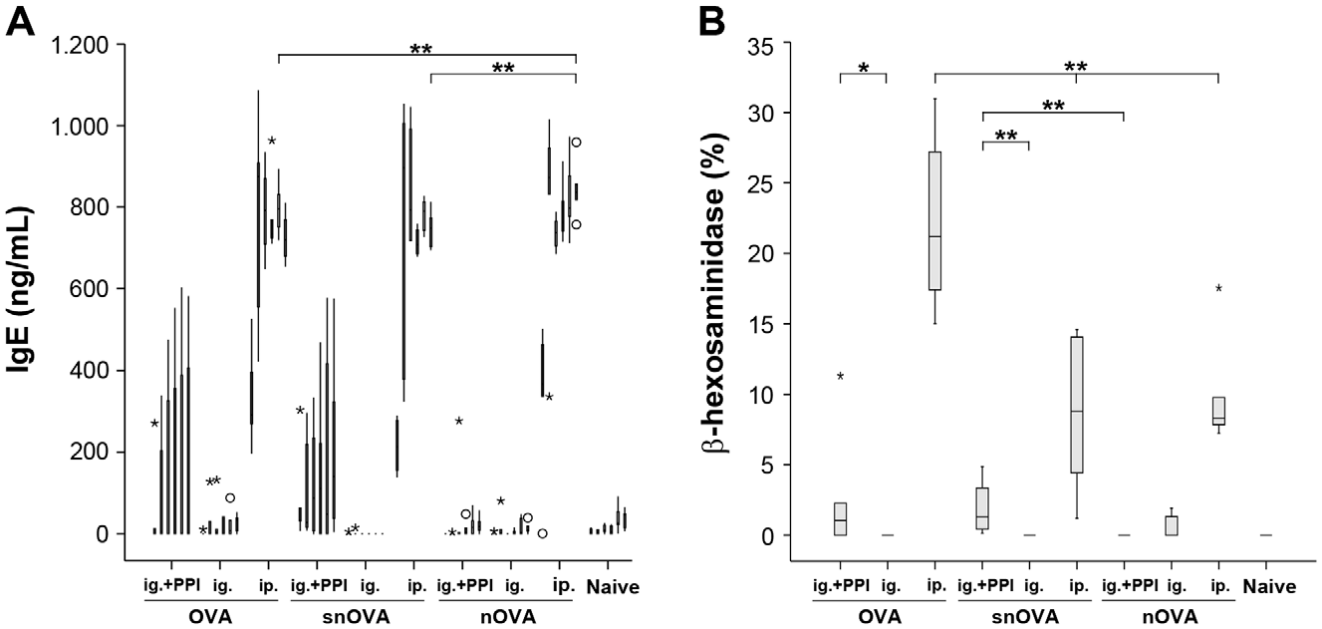
Functional IgE antibodies are detected in the acid-suppressed animals fed with OVA and snOVA and after systemic administration of OVA. (A) ELISA measurements confirmed the presence of OVA-specific IgE antibodies in the groups fed OVA and snOVA under anti-acid medication (OVA ig.+PPI, snOVA ig.+PPI) and in the ip. immunized groups (OVA ip., snOVA ip. and nOVA ip.). After systemic administration, antibody titers were significantly elevated when using nOVA, compared to OVA and snOVA (**P<0.01). Screening of murine sera from groups immunized with OVA ig., snOVA ig., nOVA ig. under concomitant anti-acid medication, nOVA ig. and from the naïve animals demonstrated only basal IgE levels. (B) The biological functionality of the detected IgE antibodies could be confirmed in an RBL assay using sera collected after the repeated immunization cycles. Again elevated levels of released mediators could be triggered upon OVA stimulations with sera of the acid suppressed animals receiving OVA or snOVA ig., as well as with sera from the ip. injected groups. The boxes represent the inner quartiles value range with the median indicated as black line. Outliers and extremes are marked with circles and asterisks. Brackets specify the groups with statistically significant differences in released mediator levels (*P<0.05, **P<0.01).

To obtain information about the allergic status of the animals, a serological screening for OVA-specific IgE antibodies was performed. ELISA measurements confirmed the pattern observed for IgG1 and IgG2a. Only animals immunized with gavages of OVA or snOVA being simultaneously acid-suppressed and in the groups immunized ip. with OVA, snOVA and nOVA developed elevated titers of specific IgE antibodies during the immunization cycles. Again, only background levels were measured in the groups being immunized orally with nOVA (Figure 3A). Interestingly, ip. injections of nOVA resulted in significantly elevated IgE titers compared to the animals being injected OVA or snOVA.

To confirm the functional relevance of the detected IgE antibodies, RBL assays using OVA as the triggering antigen were performed. In accordance with the ELISA results, mediator release could be detected only in the groups immunized with OVA or snOVA ig. under acid-suppression as well as in the groups being immunized ip. with OVA, snOVA or nOVA (Figure 3B). Highest levels of released mediators were measured in animals of the group being immunized ip. with the triggering allergen OVA.

#### Increased cytokine levels are measured in gastric tissue samples of acid-suppressed animals after oral immunization

To further analyze the immune events induced by the various treatments, cytokine levels at the site of allergy induction were evaluated in semi-quantitative RT-PCR experiments. We used gastric tissue samples from mice being immunized with anti-acid treatment via the oral route and compared the determined levels to those measured in samples from animals receiving the respective allergen without acid suppression. In line with the serological evaluations, increased levels of the Th2 cytokines IL5 and IL13, the eosinophil chemoattractants CCL11 and CCL24, as well as the inflammatory marker TNF-α were observed in the groups being fed OVA or snOVA under concomitant acid-suppression. In the animals which were orally immunized with nOVA under acid-suppression only CCL24 levels were elevated, whereas for all other measured cytokines and inflammatory markers comparable levels were detected as in tissue samples from animals being fed nOVA alone (Table 3).

**Table 3:** RT-PCR cytokine evaluation of gastric tissue samples from orally immunized animals.

	IL 5	IL13	CCL11	CCL24	TNFα
**OVA ig.+PPI**					
Mean	2.4998	2.0753	1.1825	22.4612	2.512
Lower 95% CI	0.0	0.0	0.0	9.6095	0.7776
Upper 95% CI	5.974	4.4966	2.8039	35.3130	5.8017
**snOVA ig.+PPI**					
Mean	2.6246	9.9665	1.5226	0.8021	1.8883
Lower 95% CI	1.7774	0.0	0.9745	0.0	1.3081
Upper 95% CI	7.0266	34.4637	2.0706	1.9229	2.4685
**nOVA ig.+PPI**					
Mean	1.2252	0.8637	0.9019	15.7047	0.6863
Lower 95% CI	0.0	0.0	0.0	0.0	0.5820
Upper 95% CI	2.6872	1.8387	1.9966	59.3320	0.7906

Fold increase of levels measured in animals immunized ig. with OVA, snOva and nOVA under concomitant acid suppression to animals immunized with the respective allergen ig. is presented.

#### nOVA induces significantly enhanced mediator release in RBL cells

**Figure 4:**
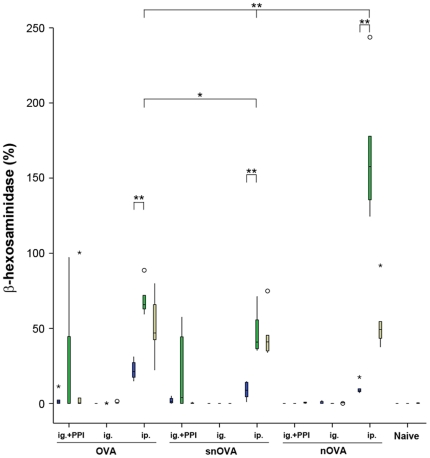
Elevated levels of released mediators are detected when triggering sensitized RBL cells with nOVA. RBL cells sensitized with the 6^th^ immune serum from the immunized or naïve animals were challenged with OVA (blue boxes), nOVA (green boxes) or nitrated Bet v1 (beige boxes). An enhanced mediator release was observed upon triggering with nOVA in RBL cells sensitized with sera of groups fed with OVA and snOVA under anti-acid medication. This elevated mediator release was also observed with sera from the positive control groups (OVA, snOVA and nOVA ip.), which additionally reacted with nitrated Bet v1. No mediator release with any of the allergens was observed in RBL cells sensitized with sera of groups immunized with OVA ig., snOVA ig., nOVA ig. under concomitant anti-acid medication, nOVA ig. and of the naïve animals, where no IgE was detected. The boxes represent the inner quartiles value range with the median indicated as black line. Outliers and extremes are marked with circles and asterisks. Brackets indicate the groups with statistical significant differences in released mediator levels (*P<0.05, **P<0.01).

RBL cells sensitized with sera from the different immunization groups were incubated with OVA (Figure 4, blue boxes), nOVA (Figure 4, green boxes) or nitrated Bet v1 (Figure 4, beige boxes). Interestingly, a significant increase of released mediators was observed in the groups positive for OVA-specific IgE (the acid-suppressed animals receiving OVA and snOVA ig. or the three groups immunized ip. with OVA, snOVA or nOVA) when nOVA was used as the triggering antigen (Figure 4, green boxes). The increase of released mediators was especially pronounced in the RBL cells immunized with sera after ip. immunizations with nOVA, where the effect even exceeded the release induced by control incubations with the detergent Triton X-100.

In the cells sensitized with sera from the ip. immunized groups an additional elevated release upon challenge with nitrated Bet v1 was observed (Figure 4, beige boxes).

#### Digestion experiments demonstrate the rapid degradation of nOVA under physiological gastric conditions

**Figure 5:**
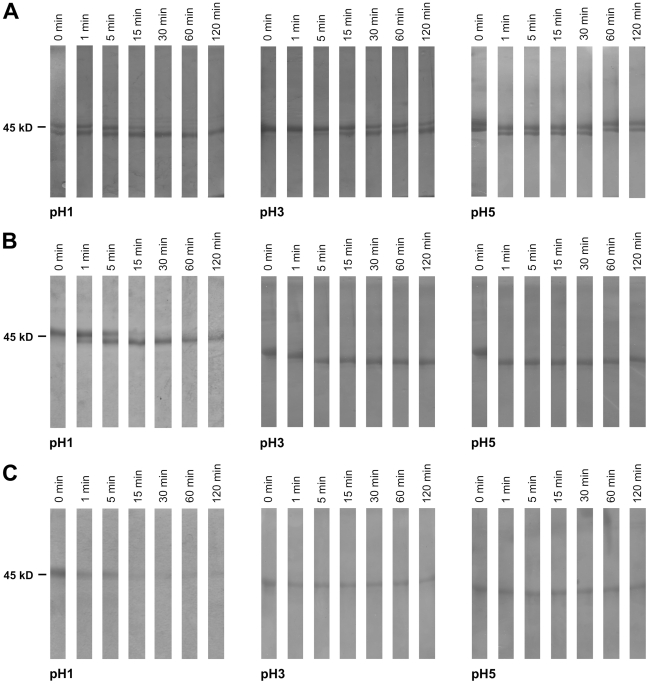
Digestion experiments reveal nitration of OVA to interfere with its resistance against gastric digestive enzymes. SGF experiments with (A) OVA, (B) snOVA and (C) nOVA reveal rapid degradation within 5 minutes only when incubating nitrated OVA with gastric enzymes at physiological gastric conditions (pH 1). At elevated pH levels (pH 3 and 5) all protein samples remained stable for up to 120 min.

Simulated gastric fluid (SGF) experiments with the immunization antigens OVA, snOVA and nOVA revealed digestion of nOVA proteins (Figure 5C) within 5 min under physiologically low pH levels (pH1). OVA (Figure 5A) and snOVA (Figure 5B) remained stable up to 120 min when being incubated with gastric enzymes at low pH. When the pH of SGF was adjusted to pH 3 and 5 simulating situations of elevated gastric pH conditions, all 3 protein preparations were not degraded during the entire incubation time of 120 min.

## Discussion

In the present study we investigated the allergenic potential of chemically nOVA in a murine model of food allergy. Interestingly, we observed a reduced sensitization capacity of nOVA upon oral antigen administration. However, systemic administration of nOVA induced high titers of biologically functional IgE antibodies. Additionally, the nitrated proteins induced a significantly higher mediator release of effector cells being sensitized with sera of allergic animals. In contrast to OVA, gastric digestion experiments revealed nOVA to be degraded within a few minutes under physiologically low pH conditions.

Our study underlines the importance of considering differences in administration routes in experimental models of food allergy. Despite the significant immunological differences [27], rodent models are crucial tools to analyze mechanisms of food allergic reactions and the allergenic potential of dietary proteins [28]. Our group has previously reported that feedings under impaired gastric digestion conditions by elevating the gastric pH results in development of food allergy [23], [24]. These findings helped to establish a mouse model of food allergy [26], reflecting the situation in allergic patients [24], [25]. This murine immunization protocol was applied in the current study to investigate the impact of food protein nitration on oral sensitization.

Chemical protein modification has been discussed to be of interest with respect to allergic reactions. Special attention has been given to the reduction of the allergic potential by chemical alterations [29]–[31] and to possible implications on the development of novel treatment strategies [32]. It has also been recognized that environmental pollutants can modify pollen allergens chemically [6] with impact on antibody recognition [7]. Nitrated Bet v1 as well as nOVA have increased allergenicity [11]. In line with these findings, we found nOVA to have enhanced triggering capacity on effector cells in our experiments. When testing with sera of allergic animals, the allergenicity of OVA proteins was enhanced if tyrosine residues were nitrated. Murine IgE antibodies specifically interacted with nOVA leading to an elevation of mediator release if immunizations were performed via the oral route. However, when animals received the OVA preparations systemically via ip. injections, an additional cross-recognition of an unrelated nitrated allergen (Bet v1) was observed even if the animals were sensitized with OVA or snOVA [11].

The results of the SGF experiments indicate that nitration of tyrosine residues interferes with the protein stability in the presence of gastric digestive enzymes. The acidic gastric milieu has been discussed to play an important role in nitration as nitrous acid was found to be formed exclusively at low pH, having then the ability to nitrate tyrosine residues of ingested proteins [33]. Nitrated proteins were demonstrated to be cleaved by the pancreatic enzyme chymotrypsin at a significantly slower rate than untreated peptides [34]. After 4 hours of chymotrypsin digestion, 40% of nitrated peptides remained stable compared to 10% of untreated peptides [7]. To our knowledge this is the first report describing that nitration enhances the digestibility of food proteins by gastric enzymes. Pepsin is known to cleave proteins preferentially at phenylalanine, tyrosine and leucine residues [35], [36]. Due to conformational changes upon nitration of tyrosine residues, amino acids interacting with pepsin might become accessible leading to an enhanced susceptibility to gastric, digestive proteases and a reduced immunogenicity if administered via the oral route. However, we have repeatedly demonstrated that our oral immunization protocol of protein feeding under concomitant acid suppression results in allergic sensitization indicated by specific IgE antibodies, leading to the development of food allergy even if food proteins were easily degraded by gastric enzymes [23], [24], [26], [37]. Thus, it might be of special interest that the most efficiently nitrated tyrosine residue within the nOVA protein (Y_107_) is part of human as well as murine IgE epitopes of ovalbumin [38], [39] and is also found in a human ovalbumin T cell epitope [40]. Using different routes of allergen application in BALB/c mice, ovalbumin epitopes were analyzed by induced antibodies revealing the tyrosine residue Y_107_ to be part of an ovalbumin epitope recognized exclusively after oral sensitization [39]. This might offer an explanation why additionally to reduced IgE levels no IgG1 or IgG2a formation was observed after oral immunizations with nOVA.

Our data demonstrate that nitration of OVA, as it might occur endogenously e.g. during inflammatory processes, results in reduced *de novo* sensitization capacity of this common egg white allergen via the oral route underlining the importance of gastrointestinal digestion in food allergy. Nevertheless, in situations where patients are sensitized and effector cells of allergy are already armed with allergen specific IgE, contact with the nitrated allergen e.g. by inhalation might result in an increased release of preformed mediators as indicated by elevated triggering capacity.

## Materials and Methods

### Antigen preparation

Lyophilized OVA (Sigma, Vienna, Austria, 98% purity) was used for all experiments. Proteins were nitrated or sham-nitrated as previously described [11]. For nitration, allergens were dissolved in PBS and nitrated using tetranitromethane dissolved in methanol (Merck, Darmstadt, Germany). Nitrated proteins were purified by size exclusion chromatography (PD-10 columns, Pharmacia, Uppsala Sweden) and the amount of 3-nitrotyrosine residues was determined by spectrophotometry at 440 nm, after dilution in 0.05 M NaOH. The spectrophotometer was calibrated with free 3-nitrotyrosine. Sham-nitration of allergens was performed using the same protocol, however without addition of tetranitromethane. Nitration of OVA was additionally confirmed by ELISA. Microtiter plates (Maxisorp, NUNC, Roskild, Denmark) were coated with 1 µg untreated, sham-nitrated or nitrated OVA per well. After blocking with TBST (Tris buffered saline with Tween-20) with 1% dried milk powder (DMP), monoclonal mouse nitrotyrosine-specific antibody (clone HM11; Invitrogen, Camarillo, CA) diluted 1:1000 in TBST/0.1% DMP were incubated overnight at 4°C. Bound antibodies were detected using peroxidase labeled goat anti-mouse IgG (Abcam, Cambridge, UK, diluted 1:5000). For detection, TMB (tetramethylbenzidine, BD Bioscience, Vienna, Austria) was added for 10 minutes and the reaction was stopped with 1.8 M H_2_SO_4_. The color reaction was measured at 450–630 nm.

### HPLC-chip-MS/MS analysis of nOVA

After nitration nOVA samples were subjected to the HPLC-chip-MS/MS analysis. The tryptic digested and desalted protein samples were analyzed with a HPLC-chip MS/MS system consisting of a nano pump (G2226A, Agilent) with 4-channel micro-vacuum degasser (G1379B, Agilent), a microfluidic chip cube (G4240-64000, Agilent) interfaced to a Q-TOF mass spectrometer (6520, Agilent; nominal mass resolution 20000 at a scan rate of 5 s^−1^), a capillary pump (G1376A, Agilent) with degasser (G1379B, Agilent), and an autosampler with thermostat (G1377A, Agilent). All modules were controlled by Mass Hunter software (version B.02.00, Agilent). A microfluidic reversed phase HPLC chip (Zorbax 300SB-C18, 5 µm particle size, 75 µm i.d., and 150 mm length) was used for peptide separation. For each chromatographic run, the solvent gradient started with 97% water with formic acid (HCOOH volume fraction 0.1%, Chromasolv, Sigma, Seelze, Germany) and 3% acetonitrile at 600 nL min^−1^. Then the acetonitrile (Chromasolv, Sigma, Seelze, Germany) content was increased to 70% within 28min. Finally, the mobile phase was reset to initial conditions within 0.1 min, and the chip was equilibrated for 2 min before the next run. The ESI-Q-TOF instrument was operated in the positive ionization mode (ESI+) with an ionization voltage of 1750 V and a fragmentor voltage of 175 V at 300°C. Fragmentation of protonated molecule ions was conducted in the auto MS/MS mode starting with a collision energy voltage of 2.5 V that was increased by 3.7 V per 100 Da. The selected m/z ranges were 300 to 2400 Da in the MS mode and 59 to 3000 Da in the MS/MS mode. The instrument settings were 4 s^−1^ for the MS scan rate and 3 s^−1^ for the MS/MS scan rate. The data output was one full mass spectrum with three fragmentation patterns per mass spectrum every 250 ms. The three highest peaks of an MS spectrum were selected for fragmentation. If the highest peaks were the same in two consecutive MS spectra, they were excluded from further MS/MS analysis (fragmentation) for the subsequent 0.08 min. Reference correction of detected ions was enabled during the whole analysis. Database searches were performed with the Spectrum Mill software (version A.03.03.084, Agilent) against the database Swiss-Prot (www.expasy.ch). Protein identification and the search for post-translational modifications including nitration were performed with the Spectrum Mill software [41].

### Animals

Eight to ten weeks old female BALB/c mice were purchased from the Institute of Laboratory Animal Science and Genetics, Medical University of Vienna, Austria. All experiments were approved by the ethics committee for animal studies of the Medical University Vienna and by the Austrian Federal Ministry of Science and Research (permission number GZ BMBWK-66.009/0001-BrGT/2007). The experiments were performed in accordance with the European Community rules of animal care.

### Immunization protocol

For investigating the effect of protein nitration on food allergy induction, our previously established murine food allergy model [26] was used. In short, animals were divided into 10 groups (n = 6 each, Table 1). Three groups were medicated intravenously with a proton pump inhibitor (PPI; omeprazole) for 3 days (on days 1–3, 15–17, 29–31, 43–45, 76–78 and 92–94). Fifteen minutes after a repeated i.v. injection of the PPI, mice were immunized orally with the different OVA preparations (0.2 mg of OVA, snOVA or nOVA per mouse) mixed with 2 mg sucralfate (Ulcogant®, Merck) on days 2–3, 16–17, 30–31, 44–45, 77–78 and 93–94. On the same days three other groups were fed the respective allergen (OVA, snOVA and nOVA) without acid suppression.

To compare different routes of exposure, six animals were immunized ip. with 2 µg OVA adsorbed to 2% aluminum hydroxide solution (1.3 µg Al(OH)_3_), another group of animals received 2 µg snOVA adsorbed to 2% aluminum hydroxide solution and a third group was injected 2((g nOVA in 1.3((g Al(OH)3 on days 3, 17, 31, 45, 78 and 94. The negative control group remained naïve. Blood samples were taken on days 0, 14, 28, 42, 56, 91 and 105 (Figure 1, Table 2).

### Evaluation of OVA specific antibodies in ELISA and RBL-assay experiments

Murine sera were screened for OVA specific antibody subclasses (IgG1, IgG2a, IgE) in ELISA. Microtiter plates (Maxisorp, NUNC) were coated with 1 µg OVA per well. After blocking with TBST/1% DMP, mouse sera diluted 1:100 for IgG1 and IgG2a and 1:10 for IgE in TBST/0.1% DMP were incubated overnight at 4°C. Bound antibodies were detected using rat anti-mouse IgG1, IgG2a or IgE (BD Biosciences, Franklin Lakes, NJ; 1:500) followed by a peroxidase labeled goat anti-rat IgG (Amersham, diluted 1:1000). For detection, TMB (BD Bioscience) was added for 15 min and the reaction was stopped with 1.8 M H_2_SO_4_. The absorbance was measured at 450–630 nm. Antibody concentrations were calculated according to standard dilution series after subtracting levels detected in pre-immune sera as background values.

To evaluate biologically active OVA specific IgE, a rat-basophil leukemia cell assay (RBL-assay) was performed [42]. RBL-2H3 cells, exclusively expressing the mouse high affinity IgE receptor FcεRI [43], were passively sensitized with murine sera diluted 1:10 and incubated at 37°C for 1 hour. After washing, 10 µg/mL OVA, nitrated OVA or 3((g/mL nitrated Bet v1 were added to the appropriate wells. The induced (-hexosaminidase release was measured in a fluorometer using 4-Methylumbelliferyl (-D-galactopyranoside (4-MUG, Sigma, Vienna, Austria) at 360–465 nm. Calculations were made by correlating measured values with (-hexosaminidase release of Triton X-100 (Sigma) stimulated cells, which was set 100%.

### RNA isolation, reverse transcription and real-time PCR analysis

After sacrifice of the animals, gastric tissue was shock-frozen in liquid nitrogen for further analysis. Total RNA was isolated from gastric tissue samples for evaluation of target gene (TNFα, IL5, IL13, CCL11, CCL24) expression by using the RNeasy RNA isolation Kit (Qiagen, Vienna, Austria), following the manufacturer's instruction. RNA concentration and purity was determined using the Nanodrop-1000 (Peqlab, Erlangen, Germany). Two µg of total RNA were transcribed into cDNA using the High Capacity cDNA Reverse Transcription Kit (Applied Biosystems, Vienna, Austria).

Expression analysis was performed by real-time PCR on a StepOne Plus real-time PCR system (Applied Biosystems). The analysis was carried out with a two step protocol starting with 20 seconds at 95°C, followed by 40 cycles of 1 second at 95°C and subsequent 20 seconds at 60°C. In all experiments duplicates were set up containing the POWER SYBR Green PCR Master Mix (Applied Biosystems) reagent. Primers were designed using “Primer Express 2.×” software (Applied Biosystems) and span, when possible, exon–intron boundaries to avoid signals from contaminating genomic DNA. The forward (fwd) and reverse (rev) primers consist of the following sequences: beta Actin fwd: cgatggtgtcactgggctc, rev: ccaccgatccacacagagtactt; CCL11 fwd: tccacagcttctattcct, rev: ctatggctttcagggtgcat; CCL24 fwd: accccagctttgaactctga, rev: aaggacgtgcagcaagatg; TNFα fwd: ctccctctcatcagttctatgg, rev: ctccacttggtggtttgctac; IL13 fwd: tattgaggagctgagcaacatcac, rev: tctgggtcctgtagatggca; IL5 fwd: tgttgacaagcaatgagacgatga, rev: ggacagtttgattcttcagtatgtc.

Based on melting curve analysis no primer–dimers were generated during the PCR amplification. For relative quantification, the expression levels of target genes were analyzed using “StepOne software 2.0” (Applied Biosystems) and normalized to the average of the house keeping gene beta-actin. The values are represented as 2∧(-ΔΔCT). For calculation of fold increase the determined expression values in animals immunized with OVA, snOVA or nOVA under gastric acid suppression were normalized to the levels derived for the groups being fed with the respective allergen alone.

### Digestion experiments

SGF was prepared with a pharmaceutical enzyme tablet (Enzynorm® forte, Pharmaselect Handels GmbH, Vienna, Austria) as previously described [44] with slight modifications: One tablet was dissolved in 100 mL 0.9% sterile sodium chloride at pH 1.0, pH 3.0 or pH 5.0. For digestion, 500 µL of SGF was incubated with 500((g OVA, snOVA or nOVA. The digestion was quenched with 0.1 N NaOH after 1, 5, 15, 30, 60 and 120 min. The effect of incubation with SGF on protein integrity was evaluated in SDS-PAGE using Coomassie brilliant blue staining.

### Statistics

Antibody titers and RBL-assay results were statistically compared using the non-parametric Mann-Whitney U test with the SPSS 18.0 program. A *P* value <0.05 was considered statistically significant.
